# Insecticide Susceptibility Status of Wild-Caught Sand Fly Populations Collected from Two Leishmaniasis Endemic Areas in Western Turkey

**Published:** 2017-03-14

**Authors:** Mehmet Karakus, Bayram Gocmen, Yusuf Özbel

**Affiliations:** 1 Department of Zoology, Institute of Science, Ege University, Bornova, Izmir, Turkey; 2 Department of Zoology, Science Faculty, Ege University, Bornova, Izmir, Turkey; 3 Department of Parasitology, Medical School, Ege University, Bornova, Izmir, Turkey

**Keywords:** Sand flies, Insecticide susceptibility, Pyrethroids, *Phlebotomus neglectus*, Turkey

## Abstract

**Background:**

In Turkey, vector control programs are mainly based on indoor residual spraying with pyrethroids against mosquitoes. No special control program is available for sand flies. Most insecticide susceptibility tests were done for mosquitoes but not for sand flies. We therefore aimed to determine the insecticide susceptibility against two commonly used insecticides; deltamethrin and permethrin, on wild-caught sand fly populations collected in two geographically separated leishmaniasis endemic areas.

**Methods:**

Insecticide susceptibility of wild-caught sand flies to deltamethrin (0.05%) and permethrin (0.75%) using ready-to use impregnated insecticide papers of WHO was investigated in 2010 based on knockdown time using standard WHO tube-test kit and procedures. Sand flies used in this study were collected from villages of Aydin (Bascayır) and Mugla (Tepecik).

**Results:**

The resistance and early resistance were detected on the sand fly population from Mugla province against deltamethrin and permethrin, respectively. However, populations from Aydin Province were sensitive to both insecticides.

**Conclusion:**

The resistance against deltamethrin and permethrin was detected on sand fly population in Mugla Province where both insecticides have been applied for long time while no resistance was found in the insecticide free area, Aydin Province. These findings can be an indicator for showing the ability for developing the insecticide resistance in sand flies. Because of the presence and dominancy of vector sand fly species of *Leishmania infantum* (*Phlebotomus neglectus*, *P. tobbi*) in both study areas, the systematic monitoring for resistance of sand fly populations and more attention are needed by the authorities involved in control programs for sand fly-borne diseases.

## Introduction

Phlebotomine sand flies (Diptera: Psychodidae) transmit *Leishmania* (Kinetoplastida: Trypanosomatidae) parasites that can cause severe, lethal clinical form, visceral leishmaniasis (VL), and moderate skin disease, cutaneous leishmaniasis (CL). In Turkey, *L. infantum* causes VL in all over Turkey and CL in East part of Mediterranean Region and transmitted by *Phlebotomus neglectus* and *P. tobbi* while *L. tropica* causes CL and transmitted by *P. sergenti* in Southeastern Region and *P. similis* in western part of Turkey (Ozbel et al. 2000, Ok et al. 2002, Toz et al. 2013).

In recent years, more than thousands of active ingredients of the pesticides are in use for insect control in many developed and developing countries as well as in Turkey (Tomlin 1997, Koçak 1998). Since 1957, insecticides have been applied heavily for pest or vector control especially in the areas where malaria cases are seen (Curtis 1962). Insecticide usage data shows that agricultural use of pesticides has reached 30000 tons/yr in Turkey (Durmusoglu et al. 2010). For this reason, monitoring of insecticide resistance is a necessary element of any medium-scale or large-scale deployment of an insecticidal intervention.

Vector control measures using insecticides have been mainly applied against mosquitoes but they are also affecting other insect vectors indirectly. Improper, disorganized and uniform usage of the insecticides for vector control has led to the development of insecticide resistance in insects as well as different vector arthropods in tropical and subtropical countries (Singh et al. 2012). The pyrethroids are the only insecticide class used for the insect control in Europe and Turkey and widespread use of a single class of insecticide increases the risk that insects (mainly mosquitoes) can develop resistance to it. The development of insecticide resistance in the insect vector has been threatened the effectiveness of these control measures (Kishore et al. 2006, Singh et al. 2012). Routine monitoring of insecticide resistance in the natural populations of vectors is necessary and helps us to detect early resistance and improve effectiveness of operational control strategies (Aizoun et al. 2014).

In Turkey, control measures against leishmaniasis include disease notification and treatment of patients, but not specific vector control. Insecticides have been mainly used for mosquito control by indoor residual spraying and therefore many studies have been conducted on insecticide resistance in the malaria vectors (Curtis 1962, Kasap et al. 2000, Lüleyap et al. 2000, Aldemir et al. 2005, Abdallah et al. 2008, Himeidan et al. 2011).

Testing of insecticide susceptibility in leishmaniasis vectors is the first important step in insecticide resistance surveillance in a particular endemic area. So far, insecticide susceptibility status of sand flies has not been studied in Turkey. Thus, the present study was undertaken to investigate the susceptibility status of Phlebotomine sand flies to two insecticides, deltamethrin and permethrin, which are currently and commonly used for insect control in Turkey.

## Materials and Methods

### Study Sites

**Fig. 1.**
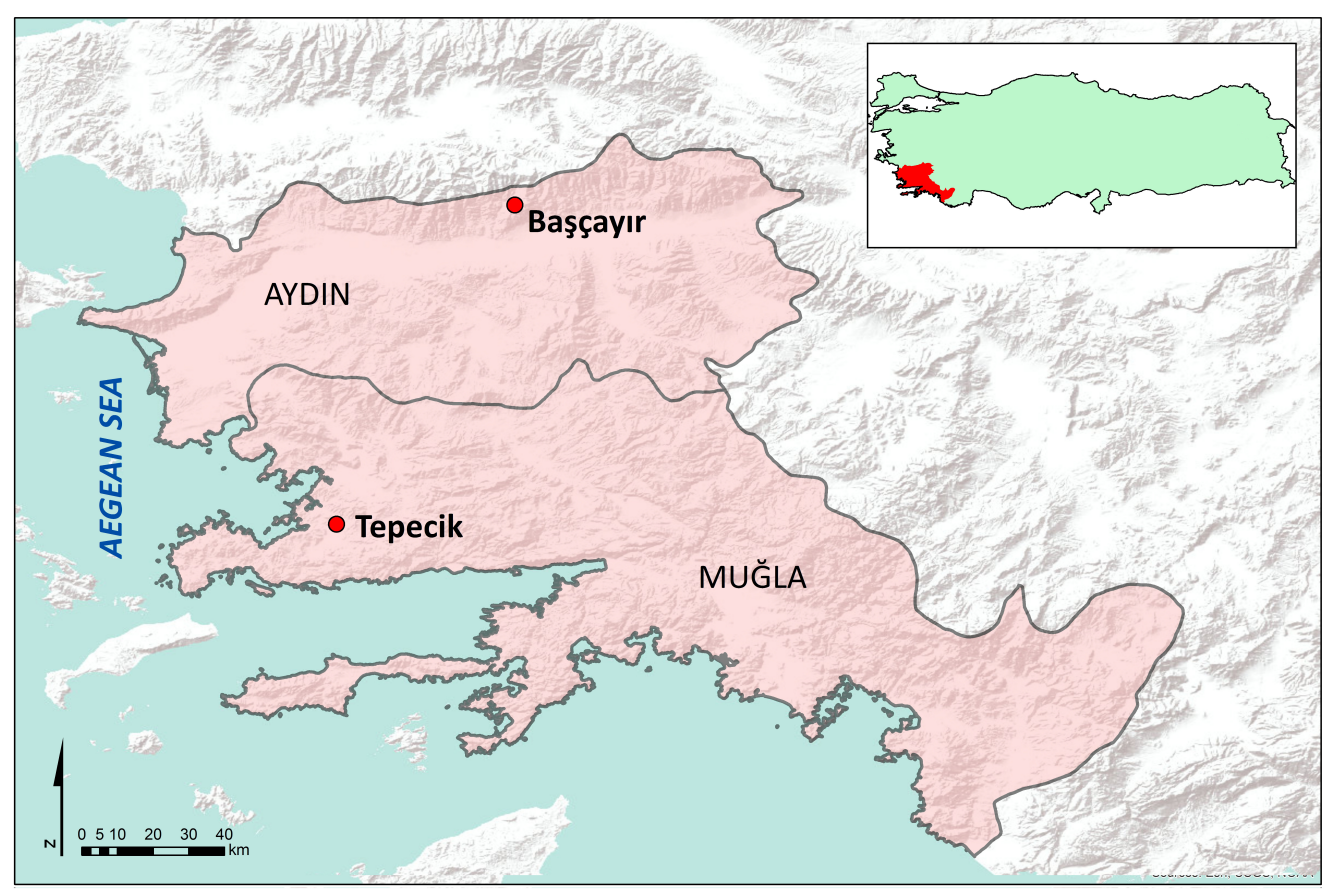
Map showing the location of both study sites where sand flies were collected

This study was conducted in two villages from two provinces where they have been subjected with different histories of insecticide exposure. Insecticides for mosquito control program have been actively used for long time in the first study area, Tepecik village in Mugla Province, while no exposure of insecticides in the second study area, Bascayir village in Aydın Province. Both study areas are endemic for cutaneous leishmaniasis in human and canine leishmaniasis in dogs, located in western part of Turkey and had similar environmental/climatic condition and geographic features (Table 1, Fig. 1).

**Table 1.** Demographic information of study sites

**Environmental characteristics of the areas studied**	**Study area 1 (Tepecik Village, Muğla)**	**Study area 2 (Bascayir Village, Aydin)**
**Latitude**	37° 08′ 06.46″ N	37° 58′ 26.74″ N
**Longitude**	27° 38′ 42.96″ E	28° 04′ 21.98″ E
**Altitude (m)**	82	359
**Population**	369	1614
**Climate type**	Mediterranean	Mediterranean
**Average temperature in summer (2010)**	28.45 °C	23.61 °C
**Average temperature in winter (2010)**	11.32 °C	11.73 °C
**Annual mean rainfall (2010)**	1050 mm	670 mm

### Sand fly Collection

Sand flies were collected using CDC light traps from both villages between June and September 2010. Totally, 20 CDC light traps were set up in each village at 7 PM. and collected at 7 AM. following day. Traps were placed mostly in animal barns. Alive sand flies from the light traps were released into a plexiglas cage by mouth aspirator and a 10% sucrose solution soaked cotton was placed on the cage. Then they were transported to the Leish-Bio-Lab in Ege University Faculty of Medicine, Izmir and kept in appropriate conditions (25±2 °C and 70±10% relative humidity).

### Insecticides and Bioassay Tests

Permethrin and deltamethrin at concentrations 0.75% and 0.05% respectively were used with an exposure time of 60 min. The choice of these two insecticides was justified by their widely use in the formulations in Turkey. WHO test-kit tubes and impregnated papers were procured from collaborating center of WHO in Malaysia.

All the susceptibility tests were done according to standard WHO testing protocols on unfed female sand flies using at least 20 specimens (not yet identified). The sand flies were transferred into the exposure tubes and were gently transferred to the holding tube after one-hour exposure period and fed with 10% sugar solution placed in the top of the holding tube. Control test tubes carrying control papers were also held parallel to each set of tests. All the tests were ignored if the mortality was higher than 20% in the control group. The test was done in five replicates for each insecticide. During these bioassays, laboratory condition was stabilized at 27±2 °C and 80±10% RH as stated on WHOPES (WHO 1981). The specimens were kept for several hours in the lab and all tests were started at between 06:00 and 07:00 PM.

The resistance status of sand fly specimens was determined according to the latest WHO criteria (WHO 2013) as follows, (a) mortality rates between 98–100% indicate full susceptibility, (b) mortality rates between 90–97% require further investigation, (c) mortality rates < 90%, the population is considered resistant to the tested insecticides.

Knockdown rates were recorded as indicated in WHOPES starting from 10 min, 15 min, 20 min, and then 10 min intervals up to 1 h for determining the exact knockdown time (KdT) of the populations, which is important for the detection of early resistance. KdT_50_, KdT_95_ and KdT_100_ values were also noted for both insecticides.

Following the testing procedures, all sand fly specimens were dissected, mounted and identified according to the keys and descriptions presented by (Perfil’ev 1968, Lewis 1987, Killick-Kendrick et al. 1991).

### Data analysis

Data analysis was made using log-probit analysis software (Probit V1.5). This software is able to calculate of KdT_50_, KdT_95_ and KdT_100_ and their confidence intervals (Finney 1971).

## Results

### Sand fly fauna of the study sites

Totally, 486 sand fly specimens used in the study were dissected, mounted and identified. Sand fly fauna of both study areas was very similar as follows: Tepecik village in Mugla Province, 3 *Phlebotomus* (64% *P. tobbi*, 30% *P. papatasi*, 5% *P. neglectus/syriacus*) and one *Sergentomyia* (1% *S. minuta*) species, Bascayir village in Aydın Province, 4 *Phlebotomus* (79% *P. tobbi*, 9% *P. neglectus/syriacus*, 6% *P. papatasi*, 2% *P. alexandri*) and 2 *Sergentomyia* (2% *S. minuta*, 2% *S. dentata)* species were found (Table 2).

**Table 2.** Sand fly species used in the study and the results of insecticide susceptibility tests

	**Insecticide Exposed area (Tepecik Village, Mugla)**	**Insecticide Free area (Bascayır Village, Aydın)**
**Number of sand fly specimen**	240	246

**Sand fly species used in the tests - Fauna (%)**		

***Phlebotomus tobbi***	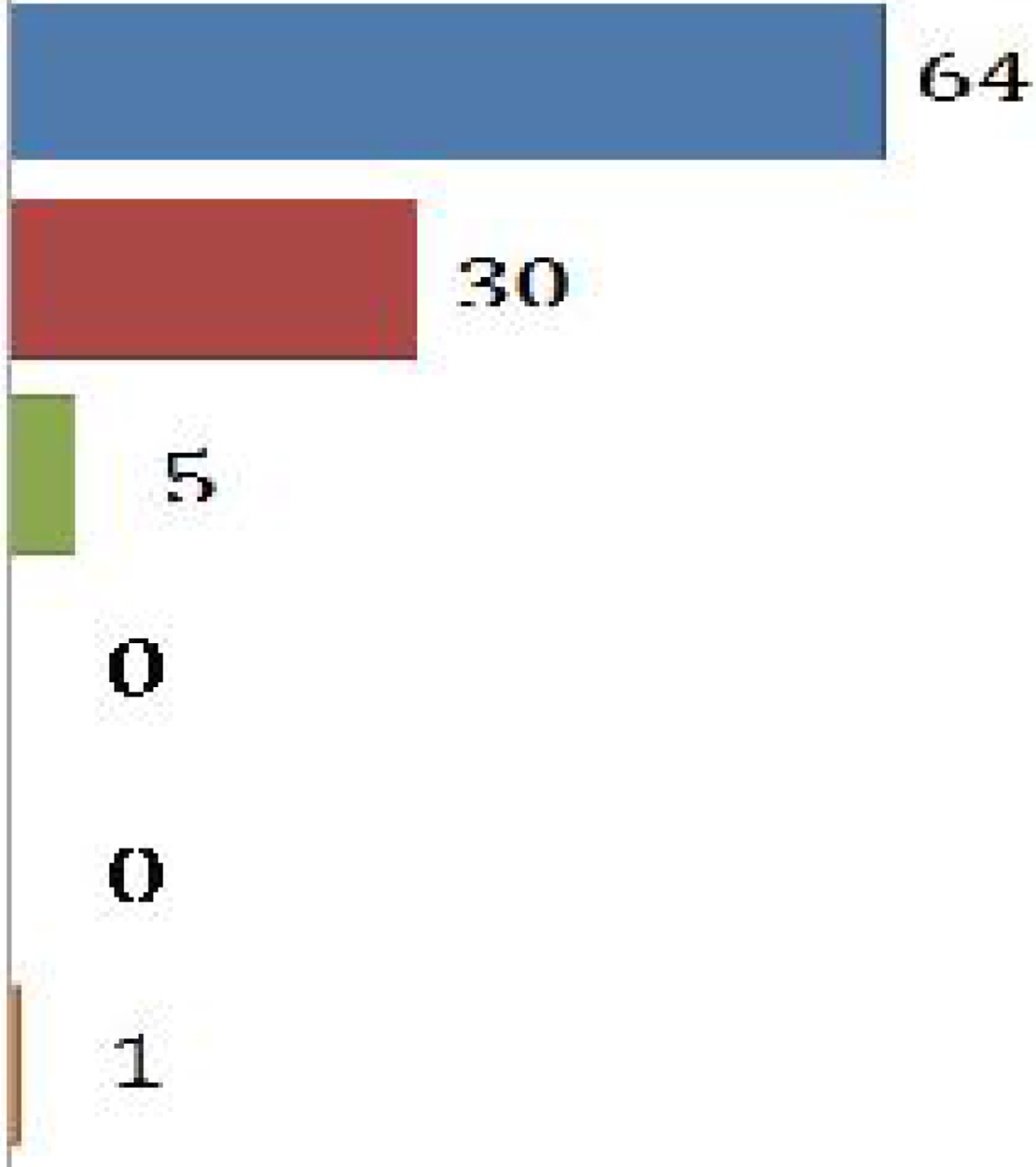	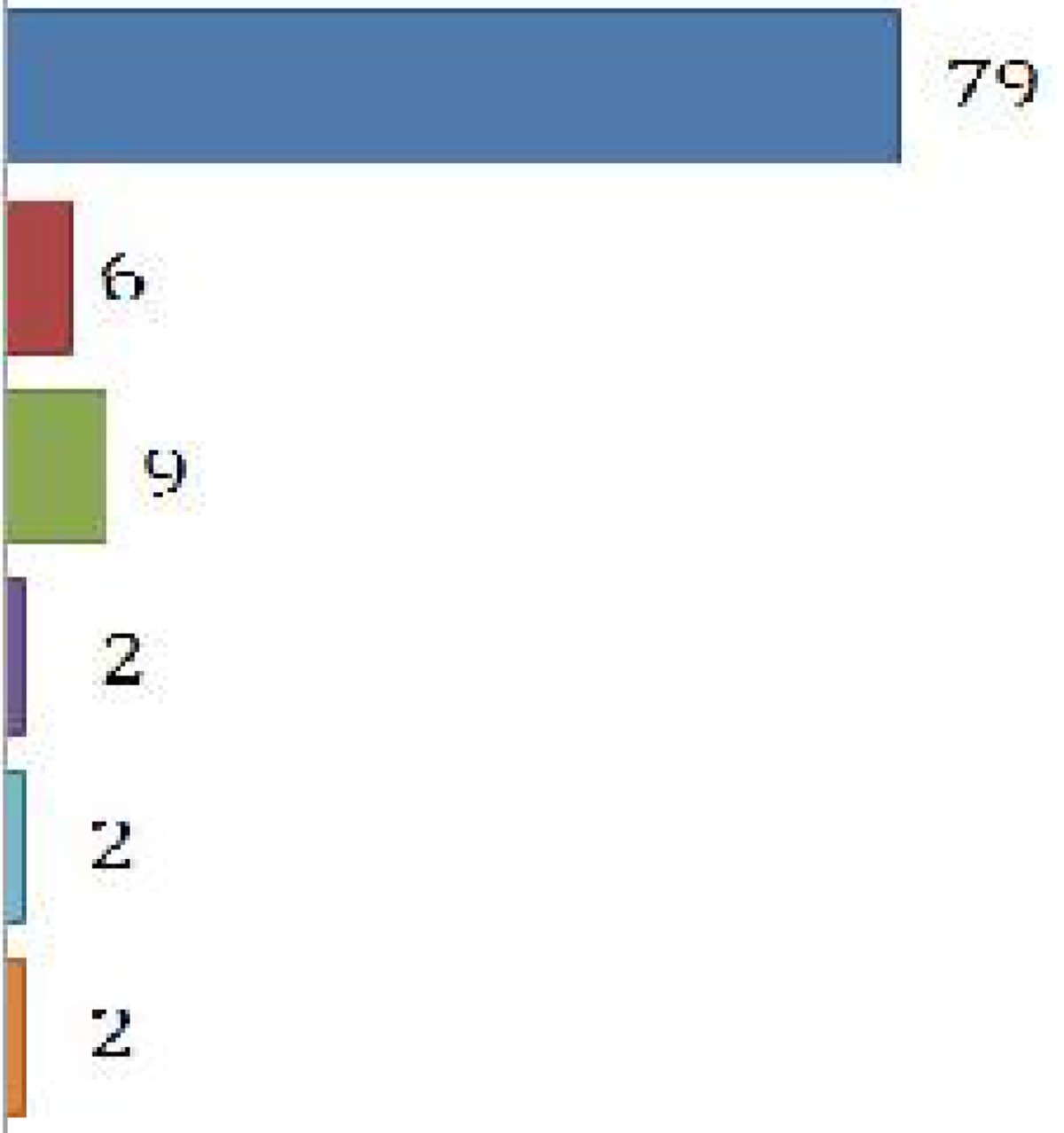
***P. papatasi***		
***P. neglectus/syriacus***		
***P. alexandri***		
***Sergentomyia dentata***		
***S. minuta***		

**Susceptibility rate (% in 24 hours) and WHO status**		

**Against deltamethrin**	90 – resistance	99 – susceptible
**Against permethrin**	93.3 – early resistance	100 - susceptible

**Observations**		

**First death**	in 1 h	in 5 min
**At the end of 24 h**	10% of the total specimens was alive but not able to fly	All dead

### Insecticide susceptibility tests

The test results were evaluated according to the WHO standards, and resistance/early resistance was detected on sand fly specimens collected in first study area (Mugla) against both insecticides while the specimens caught in the second study area (Aydin) were susceptible to both insecticides. Different death and knockdown rates were found in the sand flies from both areas. The relative susceptibilities of the two sand fly populations to tested insecticides were comparable.

### First study area (Tepecik Village, Mugla Province)

**Fig. 2.**
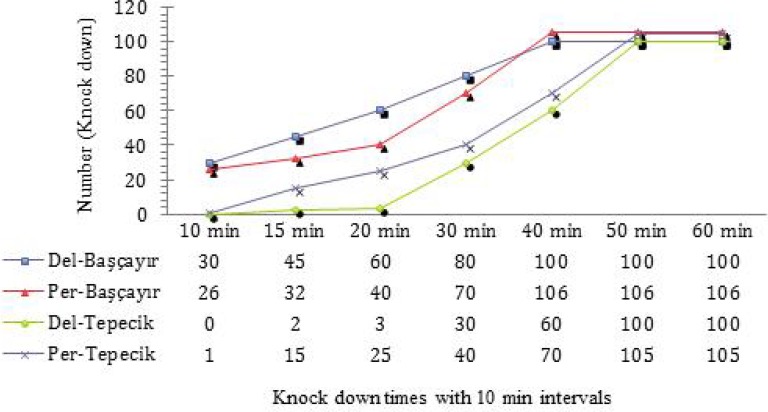
Number of knocked down sand flies and knock down times of sand flies after exposure with deltamethrin (Del) and permethrin (Per) with 10 min intervals in two areas (Bascayir and Tepecik)

For the sand fly specimens collected in Mugla Province, 90% of death rate for deltamethrin (0.05%) was noted by the end of 24 h. First knockdown effect was observed by the end of 10 min and 10 of 100 specimens was noted alive but not able to fly after 24 h (Table 2, 3, Fig. 2).

**Table 3.** The results of insecticide susceptibility tests and knockdown times of wild-caught sand flies in the study areas

**Study area**	**Insecticide**	**No. of sand flies exposed**	**Mortality rate (%)**	**KDT_5_0 min**	**KDT_9_5 min**	**KDT_10_0 min**	**WHO status**
**Tepecik Village, Muğla**	Deltametrin 0.05%	100	90	37,5	40,7	>45	T
	Permethrin 0.75%	105	93.3	34,6	36,1	>42	T
	Control	40	1				

**Bascayir Village, Aydin**	Deltametrin 0.05%	100	99	18,6	38,6	>35	S
	Permethrin 0.75%	106	100	22,4	35,9	>33	S
	Control	45	1				

**T**: Tolerance (Resistance that needs to be confirmed), **S**: Susceptible, *The knockdown rates and mortality rates were given as an average of 5 repeated tests

Same results were obtained for permethrin (0.75%) and 93.3% of death rate was noted by the end of 24 h. No knockdown effect was noted in first 10 min after exposure.

### Second study area (Bascayir Village, Aydın Province)

For the sand fly specimens collected in Aydın Province, 99% of death rate for deltamethrin was noted by the end of 24 h. Bascayir village population was found to be susceptible to deltamethrin. The first knockdown effect was observed in 3 min after exposure and 99 out of 100 specimens dead by the end of 24 h (Table 2, 3, Fig. 2).

Same results were obtained for permethrin (0.75%) and 100% of death rate was noted by the end of 24 h. Bascayir village was susceptible to permethrin (0.75%) by the terms of WHO. First knockdown effect was observed in 3 min after exposure and all specimens died in 24 h.

### Control group

One hundred sand fly specimens per study area were used for control and none of the specimens was died during the experiment except physical deaths. Abbott’s formula was not used in this study for the correction of mortality rates because of the mortality rates in all controls was always less than 5%.

### Knockdown time (KdT)

KdT50 and KdT95 knockdown times for diagnostic doses of both insecticides have calculated for sand fly populations in both study area. No significant knockdown rate for diagnostic doses of insecticides were recorded for the specimens collected in 1^st^ study area while delayed knockdown times (>45 min) were detected for the specimens collected in 2^nd^ study area (Table 3).

## Discussion

In Turkey, after *Plasmodium vivax* malaria reached the elimination level, leishmaniasis, especially cutaneous form, remained most serious and important vector-borne disease reported from all geographical regions of the country. According to Turkish Ministry of Health official reports, more than 46.000 CL cases were reported between 1990 and 2010 (Gürel et al. 2012) and 2200 CL and 40 VL cases were recorded in the year 2013 (Ministry of Health of Turkey 2012).

The control of leishmaniasis is only based on free treatment of VL and CL cases in Turkey. However, indoor residual spraying, using pyrethroids have been mainly used for mosquito or malaria control programs in most of the regions in the country and it affects other insects including sand flies. The insecticide application activity can be disparate in provinces from one to another according to the endemicity of vector-borne diseases and/or importance for tourism. The present study was undertaken because no data were available about the susceptibility of sand flies to WHO recommended insecticides in Turkey. Here, we reported the results of the first preliminary study on insecticide susceptibility of wild-caught sand fly populations in two leishmaniasis endemic areas with different history of insecticide application activities.

The principle of the WHO bioassay is to expose insects to a given dose of insecticide for a given time to assess susceptibility or resistance. In Turkey, insecticide susceptibility tests were mainly done for different mosquito species but no single study was conducted on Phlebotomine sand flies. For this reason, we compared our findings with different bioassays, conducted on sand flies in different countries. Although, deltamethrin 0.05% is the discriminating concentration given for *Anopheles*, it is not obvious to extrapolate this to sand flies. In Brazil, bioassays with 0.05% deltamethrin were used and a clear difference between the insecticide susceptibility of two sand fly populations was observed (Alexander et al. 2009). In that study, the sand fly population with no history of previous insecticide exposure, 25 min was determined as LT_50_ and all sand flies died after one hour. In the sand fly population exposed to sand fly control measures using pyrethroids since long time, LT_50_ was significantly higher (40 min) and the mortality was only 62% after 1 h (Alexander et al. 2009).

There are several studies for determining insecticide susceptibility of *P. papatasi* populations, main vector of *L. major* causing CL in a wide geographical area in the Old World (Afshar et al. 2011, Faraj et al. 2012, Hassan et al. 2012, Saeidi et al. 2012). In Sudan case, *P. papatasi* was sensitive (KDT_95_: 20.16 min) to permethrin in Rahad Game Reserve and White Nile areas while it was highly resistant (KDT_95_: 193.93 min) in Surogia Village (Hassan et al. 2012). The LT50 value of deltamethrin (0.05%) against *P. papatasi* populations in Iran was recorded as 13.6 min (Afshar et al. 2011). In the present study, *P. papatasi* was representing 5% among wild-caught sand flies but in general, KDT values for both insecticides were higher in the sand fly population from Mugla Province. In particular, deltamethrin and permethrin provided 100% knockdown after 45 min in Mugla Province while it was 35 min in Aydin Province. The identification of sand flies indicated that the dominant species was *P. tobbi* in two study areas. *P. tobbi* is one of the proven vectors of *L. infantum* in Turkey (Svobodová et al. 2009) as well as in the Old World. Our results of knockdown times reveal that *P. tobbi* has resistance that needs to be confirmed especially for Mugla Province.

Prolonging death times and knockdown times are the strongest evidence of upcoming resistance on the insecticides and insect populations with extended KdT values are more prone to develop resistance to insecticides that used in the area (Martinez-Torres et al. 1998, Chandre et al. 1999, Kamgang et al. 2011). Our results clearly showed that KdT values (Table 3) of the sand fly population from Mugla Province are much longer than Aydin population and therefore the sand fly populations in former area was more prone to develop resistance in compare to latter. Upcoming resistance can be explained by long exposure to insufficient or ineffective doses of insecticides. Short KdT values of sand fly population in Aydın Province supports the idea that these sand flies were not exposed to pyrethroid-based insecticides previously.

The ready-to use impregnated insecticide papers of WHO have been used to test the susceptibility mainly in mosquitoes as well as sand flies (Hassan et al. 2012). The results of the tests at different durations of exposure indicated that wild-caught sand fly populations from Aydin province were fully susceptible to both insecticides used, whereas the early resistance was detected in the population of Mugla Province. Developing resistance to permethrin and deltamethrin in Mugla population can be attributed to long time usage of insecticides for mosquito control purposes.

## Conclusion

The early resistance against deltamethrin and permethrin were detected on sand fly population in Mugla Province where both insecticides have been applied for long time while no resistance was found in the insecticide free area, Aydin Province. These findings can be an indicator for showing the ability for developing the insecticide resistance in sand flies as also pointed out in previous studies. Because of the presence and dominancy of vector sand fly species for *L. infantum* in both study areas, the systematic monitoring for resistance of sand fly populations needs to be accepted as a public health issue. These results clearly pointed out the more attention are needed by the authorities involved in control programs for sand fly-borne diseases. Another important point is the needs create devices and guidelines (by WHO or expert committee) for applying insecticide susceptibility tests using sand flies because of the tubes prepared for mosquitoes are not actually fit for sand flies.
